# Systematic Review and Meta-Analysis on the Impact of Atrial Fibrillation on Outcomes in Patients With Inflammatory Bowel Disease

**DOI:** 10.7759/cureus.63795

**Published:** 2024-07-04

**Authors:** Gaurang H Suhagiya, Insha H Hamid, Demeke E Agago, Muhammad Arsalan, Mayankkumar D Dhakecha, Sandipkumar S Chaudhari, Calvin R Wei, Neelum Ali

**Affiliations:** 1 Medicine, Jiangsu University, Zhenjiang, CHN; 2 Physiology, Government Medical College (GMC) Srinagar, Srinagar, IND; 3 Medicine, Dilchora Hospital, Dire Dawa, ETH; 4 Medicine, Lady Reading Hospital-Medical Teaching Institute (LRH-MTI), Peshawar, PAK; 5 Medicine, Government Medical College and Hospital, Surat, Surat, IND; 6 Cardiothoracic Surgery, University of Alabama at Birmingham, Birmingham, USA; 7 Medicine, University of North Dakota School of Medicine and Health Sciences, Fargo, USA; 8 Research and Development, Shing Huei Group, Taipei, TWN; 9 Medicine, University of Health Sciences, Lahore, PAK

**Keywords:** systematic review and meta analysis, myocardial infarction, mortality, inflammatory bowel disease, atrial fibrillation

## Abstract

Inflammatory bowel disease (IBD) is a chronic inflammatory condition affecting the gastrointestinal tract, often leading to symptoms like abdominal pain and diarrhea. Given the increasing evidence linking systemic inflammation to atrial fibrillation development, investigating IBD as a potential risk factor for atrial fibrillation becomes imperative. This meta-analysis aims to evaluate the impact of atrial fibrillation on inpatient outcomes, resource utilization, and length of hospital stays among IBD patients. Following the Preferred Reporting Items for Systematic Review and Meta-Analysis (PRISMA) 2020 guidelines, a systematic literature search was conducted across multiple databases, including Embase, PubMed, Scopus, and Web of Science, from the inception of databases to June 5, 2024. Eligible studies included prospective or retrospective studies with definitive diagnoses of ulcerative colitis, Crohn's disease, or IBD, demonstrating the influence of atrial fibrillation. Data were extracted, and quality assessment was performed using the Newcastle-Ottawa Scale. The meta-analysis comprised 842,149 IBD patients, with 71,221 having atrial fibrillation. Pooled analysis revealed a significant association between atrial fibrillation and heightened all-cause mortality risk (risk ratio (RR): 1.42, 95% confidence interval (CI): 1.16 to 1.74, p<0.01). However, no significant differences were observed in the incidence of acute myocardial infarction, acute kidney injury, or acute respiratory failure between patients with and without atrial fibrillation. IBD patients with comorbid atrial fibrillation face higher mortality rates, potentially due to systemic inflammation, thromboembolism risks, polypharmacy, and the complexities of managing both conditions concurrently. Early identification and integrated management of atrial fibrillation in IBD patients are crucial to improving outcomes. Larger, multi-center studies are needed to explore the underlying mechanisms and develop tailored treatment strategies.

## Introduction and background

Inflammatory bowel disease (IBD) encompasses chronic inflammatory conditions like Crohn's disease and ulcerative colitis, affecting the gastrointestinal tract and often leading to symptoms like abdominal pain and diarrhea [1]. It includes Crohn's disease (CD) and ulcerative colitis (UC). CD is characterized by chronic inflammation of the digestive tract, causing various symptoms. UC is an inflammatory bowel disease affecting the colon and rectum, leading to symptoms such as abdominal pain and bloody diarrhea [1]. Globally, both the incidence and prevalence of IBD are increasing; the condition usually peaks between the ages of 50 and 70, with the maximum occurrence occurring between the second and fourth decades of life [2]. 

Atrial fibrillation prevalence and incidence are increasing worldwide. The Framingham Heart Study reported a threefold rise in atrial fibrillation prevalence over the past 50 years [3]. In the United States alone, at least three to six million people currently have atrial fibrillation, with projections reaching up to 16 million by 2050 [4]. Various inflammatory markers, including C-reactive protein, tumor necrosis factor-α, and interleukins 2, 6, and 8, have been linked to atrial fibrillation. There is substantial evidence connecting inflammation to the onset and maintenance of atrial fibrillation [5]. Similar to other cardiac arrhythmias, atrial fibrillation is closely associated with increased risks of cardiovascular complications, which lead to reduced quality of life, disability, higher healthcare costs, and elevated mortality rates [6]. Recently, inflammation has been recognized as a pathogenic factor in the development of atrial fibrillation [7]. Numerous cardiovascular disorders, especially coronary atherosclerosis, are related to inflammation, with cytokines playing a role in plaque rupture and thrombus formation, ultimately resulting in myocardial infarction [8]. 

Given the increasing evidence that links systemic inflammation to the development of atrial fibrillation, it is logical to investigate inflammatory bowel disease (IBD) as a potential risk factor for atrial fibrillation. The co-occurrence of atrial fibrillation and IBD can potentially worsen clinical outcomes, leading to increased disability, higher healthcare utilization, medical expenses, and mortality rates. Despite this potential link, there is a lack of comprehensive data on how atrial fibrillation affects inpatient outcomes, resource utilization, and length of hospital stays among IBD patients. No meta-analytic studies have systematically explored the impact of atrial fibrillation on the inpatient outcomes of IBD patients, especially those prone to frequent hospitalizations. This meta-analysis aims to fill this gap by synthesizing existing research to evaluate these critical parameters and related outcomes. 

## Review

Methodology 

The “Preferred Reporting Items for Systematic Review and Meta-Analysis (PRISMA) 2020” [9] guidelines and the Cochrane criteria were followed in the conduct and reporting of this systematic review. 

Study Search and Selection 

Systematic literature searches were carried out on databases including Embase, PubMed, Scopus, and Web of Science from the inception of databases until June 5, 2024. Predefined medical subject heading (MeSH) terms were used in addition to “OR” and "AND." The following search terms were utilized: “inflammatory bowel disease” OR “Crohn’s disease” OR “colitis, ulcerative” AND “atrial fibrillation” AND “all-cause mortality” OR “myocardial infarction” (Appendix A). We did not put any restrictions on the language of the studies or the year of publication. After a thorough screening process, every study was transferred to the Endnote 2020 library (X9). A manual review was done to get rid of duplicates. An abstract and titles were reviewed by two reviewers (MA and MD). In cases of disagreement over which research should be included, the senior author arbitrated. 

Studies having an age group greater than eighteen, observational studies (prospective or retrospective studies), with a definitive diagnosis of ulcerative colitis (UC), Crohn's disease (CD), or IBD, and research demonstrating the influence of atrial fibrillation were all considered for inclusion. Excluded research included animal tests, review articles, investigations, meta-analysis, review studies, case reports, and case series. Additionally, studies on patients under the age of 18, studies with a combination of autoimmune illnesses, and studies showing unfavorable cardiovascular outcomes from other causes were also excluded.

Data Extraction 

From every eligible study, we retrieved data on the first author, publication year, study region, study subjects, sample size, and risk ratio (RR) (adjusted and unadjusted) with a 95% confidence interval (CI) for outcome variables. Two reviewers (MD and SS) who separately extracted the data from each included study arbitrated disagreements by reaching a consensus. 

Quality Assessment 

The included records were assessed using the Newcastle-Ottawa Scale (NOS) [10], a tool specifically created for observational and non-randomized research. It evaluates a study's quality using a star system (from zero to nine) based on three domains: outcome/exposure (three stars), comparability (two stars), and selection (four stars). An article's quality is indicated by its star rating: seven to nine stars indicate good quality, four to six stars indicate moderate quality, and one to three stars indicate low quality. 

Statistical Analysis 

Data analysis was performed using RevMan Version 5.4.1. The relationship between atrial fibrillation and outcomes, including all-cause mortality, myocardial infarction, acute kidney failure, and acute respiratory failure, in patients with IBD was compared using a risk ratio (RR) with a 95% confidence interval (CI). To adjust for confounding variables, we used the adjusted risk ratio where available. If adjusted ratios were not available, we used unadjusted ratios. A P-value less than 0.05 was considered significant. Heterogeneity was reported in I-square. In cases of high heterogeneity, i.e., I-square, more than 50% were considered to be high heterogeneity. We performed a sensitivity analysis by removing one study at a time. 

Results 

**Figure 1:**
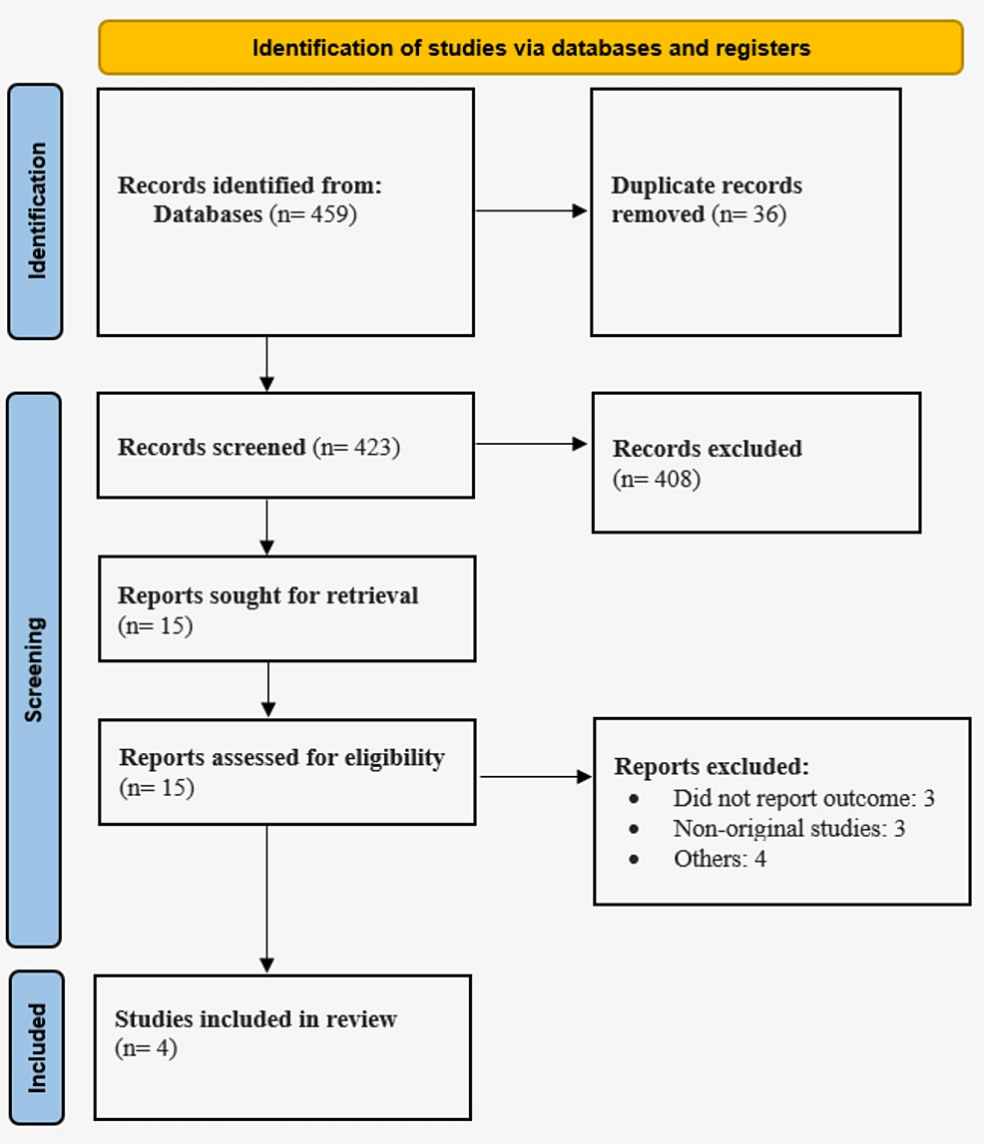
PRISMA flowchart showing study selection process PRISMA: Preferred Reporting Items for Systematic Review and Meta-Analysis

Using the initial search strategy, 459 possibly relevant articles were found in the three electronic databases, as shown in Figure 1. Thirty-six duplicates were eliminated. We found 15 records by reading the abstracts and titles; after reading the entire contents, we eventually included four articles [11-14]. Table 1 displays the features of the studies that were included. The meta-analysis comprised 842,149 IBD patients in total. Atrial fibrillation was present in 71,221 out of 842,149 IBD patients. Table 2 displays the results of the NOS quality evaluation, which indicated that three studies were of good quality. 

**Table 1:** Characteristics of studies included in this review NR: not reported

Author ID	Year	Study Design	Region	No. of Patients (n)	Atrial Fibrillation (n)	Mean Age (Years)		Males (n)	
						Atrial Fibrillation	Control	Atrial Fibrillation	Control
Kichloo et al. [11]	2021	Retrospective	United States	92055	3900	70.9	45	1775	42931
Lodhi et al. [12]	2021	Retrospective	United States	8066	677	NR	NR	NR	NR
Mahfouz et al. [13]	2022	Retrospective	United States	27165	2045	71	49	39	24
Rahman et al. [14]	2021	Retrospective	United States	714863	64599	73.2	50.8	30814	291318

**Table 2:** Quality assessment of included studies

Author ID	Selection	Comparison	Assessment	Overall
Kichloo et al. [9]	3	2	3	Good
Lodhi et al. [10]	4	2	3	Good
Mahfouz et al. [11]	2	2	2	Fair
Rahman et al. [12]	4	2	2	Good

Effect of Atrial Fibrillation on Mortality in Patients With IBD 

**Figure 2:**
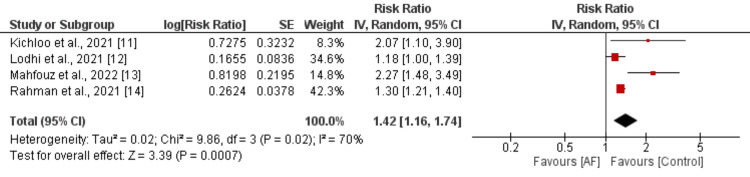
Effect of mortality on the risk of all-cause mortality in patients with IBD AF: atrial fibrillation; IBD: inflammatory bowel disease References [11-14]

Four studies examined the correlation between atrial fibrillation and mortality in patients with inflammatory bowel disease (IBD), with findings depicted in Figure 2. The pooled analysis revealed a significant association between atrial fibrillation and heightened all-cause mortality risk (RR: 1.42, 95% CI: 1.16 to 1.74, p-value<0.01). However, considerable heterogeneity was observed among the study outcomes (I-square: 70%). Sensitivity analysis was conducted, removing one study at a time, and the results are detailed in Table 3. We found that after removing the study conducted by Mahfouz et al., sensitivity reduced from 70% to 40%, owing to the fact that this study included IBD patients undergoing colectomy.

**Table 3:** Sensitivity analysis RR: risk ratio; CI: confidence interval

Study ID	RR	95% CI	I-square
Kichloo et al., 2021 [11]	1.37	1.12 to 1.67	74%
Lodhi et al., 2021 [12]	1.73	1.12 to 2.86	76%
Mahfouz et al., 2022 [13]	1.28	1.13 to 1.45	40%
Rahman et al., 2021 [14]	1.69	1.02 to 2.79	80%

*Effect of Atrial Fibrillation on Secondary Outcomes in Patients With IBD* 

We assessed the effect of atrial fibrillation on secondary outcomes, including myocardial infarction, acute kidney injury, and acute respiratory failure, in patients with IBD, and the results are shown in Table 4. The risk of acute myocardial infarction was not significantly different between patients with and without atrial fibrillation (RR: 1.01, 95% CI: 0.87 to 1.17). Similarly, the risk of acute kidney injury was also not significantly different between the two groups (RR: 1.01, 95% CI: 0.93 to 1.09). Lastly, the risk of acute respiratory failure was also not significantly different between the two groups (RR: 1.32, 95% CI: 0.87 to 2.00). 

**Table 4:** Secondary outcome analysis RR: risk ratio; CI: confidence interval

Outcomes	RR	95% CI
Myocardial infarction	1.01	0.87 to 1.17
Acute kidney injury	1.01	0.93 to 1.09
Acute respiratory failure	1.32	0.87 to 2.00

Discussion 

The key findings of this meta-analysis are as follows: IBD patients with comorbid atrial fibrillation (AFIB) have higher mortality rates compared to those without AFIB. However, there were no significant differences in the incidence of acute myocardial infarction, acute kidney injury, or acute respiratory failure between the two groups. IBD patients are at a higher risk of developing AFIB, and the presence of both conditions can result in worse outcomes and more frequent hospitalizations [15]. To our knowledge, this is the first meta-analysis to evaluate the impact of atrial fibrillation on outcomes in patients with IBD. 

Atrial fibrillation is a multifaceted condition with various underlying mechanisms. In this meta-analysis, the prevalence of atrial fibrillation is found to be 8.47%, close to the 11.3% reported by Pattanshetty et al. [13] among 141 IBD patients and notably higher than the 0.95% observed in the general population by Go et al. [16]. Several studies indicate that inflammation plays a key role in the development of atrial fibrillation [17]. For instance, Frustaci et al. identified inflammatory changes in atrial tissues in patients with isolated persistent atrial fibrillation, supporting the link between inflammation and atrial fibrillation [18]. 

Patients with IBD who also have atrial fibrillation face an increased risk of mortality due to several interconnected factors. Firstly, IBD is characterized by chronic inflammation of the gastrointestinal tract, leading to systemic inflammation, which is a known risk factor for the development and progression of atrial fibrillation [19]. Elevated levels of inflammatory markers, such as C-reactive protein (CRP), common in IBD, can exacerbate atrial fibrillation, contributing to a more complicated disease course [20]. Secondly, atrial fibrillation itself brings significant risks, including thromboembolism and stroke, as well as heart failure, all of which can severely impact mortality [19]. The requirement for anticoagulation therapy in atrial fibrillation patients further complicates the situation for those with IBD, as these medications increase the risk of gastrointestinal bleeding, a major concern in IBD management [21]. Additionally, the simultaneous management of both conditions often leads to polypharmacy, heightening the risk of adverse drug interactions and side effects [22]. These combined factors underscore the complex interplay between IBD and atrial fibrillation, resulting in a significantly higher mortality risk for patients dealing with both conditions. 

Furthermore, the link between higher mortality in IBD patients and atrial fibrillation suggests the need for screening for atrial fibrillation in this group. Early identification of atrial fibrillation and the subsequent application of appropriate treatments could lead to better outcomes for patients with IBD. 

Study Limitations 

The present meta-analysis has several limitations. Firstly, it included only four studies, which limits the generalizability and robustness of the findings. Additionally, all the included studies were observational, making them susceptible to biases such as selection bias, where the study population may not represent the general IBD and AF population, and confounding bias, where other variables may influence the observed outcomes. To mitigate these biases, the meta-analysis used adjusted risk ratios, which account for potential confounders, thereby providing a more accurate estimate of the effect of atrial fibrillation in patients with inflammatory bowel disease. 

The findings of this meta-analysis highlight the critical need for heightened clinical awareness and monitoring of atrial fibrillation in patients with IBD. Given the increased mortality risk, healthcare providers should prioritize early detection and integrated management of atrial fibrillation in IBD patients. Future research should focus on larger, multi-center studies to confirm these findings and explore the underlying mechanisms linking IBD and atrial fibrillation. Additionally, investigating the efficacy of tailored treatment strategies to manage both conditions concurrently could improve patient outcomes. This study underscores the importance of comprehensive care approaches in managing complex, coexisting conditions. 

## Conclusions

In conclusion, this meta-analysis reveals that inflammatory bowel disease patients with comorbid atrial fibrillation face a significantly higher risk of mortality compared to those without atrial fibrillation. Given the intricate interplay between these two conditions, characterized by systemic inflammation, and the challenges of managing multiple complex diseases concurrently, early identification and integrated management of atrial fibrillation in IBD patients are crucial. Healthcare providers should prioritize screening for atrial fibrillation and implement tailored treatment strategies to optimize outcomes for these high-risk patients. Larger, multi-center studies are warranted to further elucidate the underlying mechanisms and explore targeted interventions that address the unique needs of this patient population.

## Appendices

Appendix 1

Search Strategy for PubMed

(("Inflammatory Bowel Diseases"[Mesh] OR "Inflammatory Bowel Disease*" OR "IBD" OR "Crohn Disease"[Mesh] OR "Crohn*" OR "Colitis, Ulcerative"[Mesh] OR "Ulcerative Colitis" OR "UC") AND ("Atrial Fibrillation"[Mesh] OR "AF" OR "A-fib") AND ("Mortality"[Mesh] OR "Death*" OR "Survival" OR "Myocardial Infarction"[Mesh] OR "Heart Attack" OR "MI"))
